# Satisfaction and Concerns with Telemedicine Endocrine Care of Patients with Cystic Fibrosis

**DOI:** 10.1089/tmr.2021.0053

**Published:** 2022-03-21

**Authors:** Rahat Ahmed, Margaret Greenfield, Christopher P. Morley, Marisa Desimone

**Affiliations:** ^1^ Division of Endocrinology, Diabetes and Metabolism, State University of New York at Upstate Medical University, Syracuse, New York, USA.; ^2^ Department of Public Health and Preventative Medicine, State University of New York at Upstate Medical University, Syracuse, New York, USA.

**Keywords:** telemedicine, telehealth, cystic fibrosis, cystic fibrosis-related diabetes

## Abstract

**Introduction:**

Patients with chronic health conditions are at high risk for severe COVID-19 infections, making telemedicine for patients with cystic fibrosis (CF) and cystic fibrosis-related diabetes (CFRD) particularly relevant. There are limited data regarding provider perspectives on caring for patients with CF using telemedicine, particularly for those with CFRD.

**Methods:**

Surveys were administered to patients with CF (with and without CFRD) and to adult and pediatric endocrinologists who specialize in CF. Data were collected using Research Electronic Data Capture; *t*-tests were used to compare total mean scores of Likert scale questions. The differences in responses were performed using one-way analysis of variance followed by Tukey's Honest Significant Difference test. Variables were assessed for normality and we performed the Mann–Whitney test. No change in the results of the hypothesis test was found. All results were analyzed using SPSS version 27.

**Results:**

Eighteen patients (*n* = 9 CFRD) and 21 providers responded. Both groups reported high satisfaction with telemedicine overall (83.3%; 71.4%), convenience (94.4%; 85.7%), and adequate time during the visit (94.4%; 76.2%), and the majority would recommend telemedicine to others (94.4%; 95.2%). Lack of in-person examination components was of more concern to providers than patients: height/weight (*p* < 0.001), vitals (*p* < 0.001), and glycated hemoglobin (*p* < 0.001). There was no difference in provider perception in treatment of CFRD compared to type 1 diabetes (T1D). Common themes of open-ended questions included ease in attending telemedicine appointments (patients) and decrease in “no shows” (providers).

**Discussion:**

Patient and provider satisfaction with telemedicine was high. The lack of typical components of face-to-face visits was more concerning for providers when compared to patients. Provider concern regarding lack of components specific to diabetes was similar regarding CFRD and T1D.

## Highlights

- Both patients and providers conveyed a high degree of overall satisfaction with telemedicine.
- Providers showed a higher degree of concern when it came to the lack of physical examination components compared to patients.
- Providers felt that telemedicine lowered appointment “no shows” or cancellations.
- Patients felt telemedicine visits were convenient to attend since travelling was not required and lowered their risk of getting infections.

## Introduction

Cystic fibrosis (CF) is a genetically inherited disease that affects multiple organs. Outcomes of people with CF are improved when managed by specialty centers; however, not all patients have access to such care.^1–3^ Telemedicine is one strategy that has been used to improve patient access to specialty services.^4^

Telehealth is a method of health care delivery, which connects individuals and health care providers with the use of technology. This allows the patients and providers to connect with video, telephone, or chat-based interactions; remote patient monitoring and asynchronous transmission of patient data for later review can also be used.^5^ Telemedicine has been used since the 1960s for monitoring the health of astronauts by National Aeronautics and Space Association.^6^ Since its first adoption, the use of telemedicine has greatly expanded to multiple different health conditions and patient populations.

There are 133 Cystic Fibrosis Foundation accredited centers in the United States and it is recommended that patients with CF visit their center at least four times per year for routine monitoring and care.^7^ In an op-ed article from 2020, the president of the CF foundation Dr. Michael Boyle noted that 10% of patients with CF miss appointments due to transportation and cost, and that telemedicine is one modality that could be leveraged to assist in care delivery.^8^ The CF foundation actively encouraged telemedicine visits in 2020 by providing over 10,000 home spirometers to patients.^9^ Home spirometry measurements and symptoms scoring methods are already used to assess pulmonary exacerbations.^10^ However, CF affects more than just the lungs. It can cause multiple endocrine disorders such as diabetes, bone disorders, and hypogonadism.^11^

Telemedicine has been used to prevent hospitalization in pulmonary disorders such as chronic obstructive pulmonary disease and asthma; however, it has not been widely studied in the CF population.^10^ A 2011 systematic review of telehealth applications to CF patients noted that the studies have mainly been small, feasibility trials with limited external validity.^12^ At this time, there is still a lack of large studies evaluating telemedicine in the CF population. One survey of CF experienced clinicians in 2021 found that 90% had never utilized telehealth before; however, nearly all (96% on initial and 99% on follow-up survey) preferred some/most visits be completed through telehealth if it were offered in the future.^13^

Diabetes, with its related complications, is the most expensive chronic condition in the United States with an estimated $327 billion dollars in total annual cost.^14^ Telemedicine is one tool that has been used to deliver care to people with diabetes, and has been well described in the literature.^15^ Telemedicine is particularly well suited to treating patients with diabetes and can give providers access to patient biometrics such as remote blood glucose monitoring.^16,17^

The nature of diabetes care requires frequent contact between the patient and their care team for medication adjustments, education, and lifestyle changes. The convenience of telemedicine, which allows patients to get medical care without travel and its associated cost, is appealing, and patient satisfaction with telemedicine has generally been favorable.^17,18^ Telemedicine in diabetes has also been shown to be effective.^19–21^

Cystic fibrosis-related diabetes (CFRD) is a common complication of CF.^22^ As the number of adults living with CF increases, there will be an increasing need for specialist care.^23,24^ There is a shortage of endocrinologists in the United States; one study from 2014 indicated that there are only 21 adult endocrinologists for every 1 million adults.^25^ This shortage is predicted to double by 2025.^25^ Patients who live in rural areas are disproportionately affected, as there are fewer endocrinologists serving those areas.^26^

The COVID-19 pandemic caused a rapid transition from face-to-face services to telemedicine across health care systems to decrease virus transmission.^4^ Patients with chronic health conditions, including underlying lung disease and diabetes, are at high risk for severe COVID-19 infections, making telemedicine for patients with CF and CFRD particularly relevant.^27^ A survey taken in May 2020 of patients and caregivers with a diagnosed chronic condition found that 49% of the respondents engaged in telemedicine over the past 4 months. Patients with CF, lupus, and ankylosing spondylitis recorded the highest proportion of telehealth usage when compared to other chronic conditions.^28^ There are limited data regarding provider perspectives on caring for patients with CF using telemedicine, particularly for those with CFRD. This project aimed to assess CF patient and provider perspectives of telemedicine, and how it relates to barriers in the management of CFRD.

## Methods

All patients with CF seen at a single endocrinology practice in the last year were contacted by phone for a survey. Patients were excluded if they had not had at least one telemedicine visit in the past year. A total of three attempts were made to reach the patient over the phone. Patients with and without CFRD were surveyed. Patient responses were gathered by a single investigator by phone. A separate provider survey was distributed by email to a group of adult and pediatric endocrinologists who specialize in treating patients with CF and who participated in the Envision CF I and II grants from the CF Foundation.

Study data were collected and managed using Research Electronic Data Capture (REDCap) electronic data capture tools hosted at SUNY Upstate Medical University.^29,30^ REDCap is secure, web-based software platform designed to support data capture for research studies, providing (1) an intuitive interface for validated data capture; (2) audit trails for tracking data manipulation and export procedures; (3) automated export procedures for seamless data downloads to common statistical packages; and (4) procedures for data integration and interoperability with external sources.

Both the patient and provider surveys comprised multiple-choice, Likert scale, and open-ended questions. *t*-Tests were used to compare total mean scores of Likert scale questions for respondent types. The differences in responses were performed using one-way analysis of variance followed by Tukey's Honest Significant Difference test. Variables were assessed for normality and we performed the Mann–Whitney test. No change in the results of the hypothesis test was found.

All results were analyzed using SPSS v. 27. The Institutional Review Board at the State University of New York at Upstate Medical University reviewed this project and determined that it did not meet the definition of human subject research (project 1660932-1). Participants indicated their consent by completing the survey.

## Results

### Respondent characteristics

A total of 31 patients met inclusion criteria and 18 patients completed the survey. One patient did not complete the entire survey and was therefore excluded from results, four patients refused to participate, and eight patients could not be reached. Patient respondents ranged in age from 19 to 71 years (mean 38.1), 9 (50%) had CFRD, and 9 (50%) were female (Table 1).

**Table 1.** Patient Characteristics

Total, *N* (%)	18 (100)
Mean patient age (SD)	38.1 (15.8; minimum 19–maximum 71)
Patients with CFRD, *n* (%)	9 (50)
Patients gender (female), *n* (%)	9 (50)

CFRD, cystic fibrosis-related diabetes; SD, standard deviation.

Eighty-three providers were identified as either adult or pediatric endocrinologists specializing in care of patients with CF, and 21 surveys were completed. Seven (33.3%) providers cared for adult patients only, 10 (47.6%) cared for pediatric patients only, and 4 (19%) had combined adult and pediatric clinical practices. Eight respondents (38.1%) had been in clinical practice for 1–5 years, 10 (47.6%) for 6–10 years, 2 (9.5%) for 16–20 years, and 1 (4.8%) for over 20 years (Table 2).

**Table 2.** Provider Characteristics

Total, *N* (%)	21 (100)
Clinical practice, *n* (%)	
Adult only	7 (33.3)
Pediatric only	10 (47.6)
Adult and pediatric	4 (19.0)
Average years of practicing, *n* (%)	
1–5	8 (38.1)
6–10	10 (47.6)
11–15	0
16–20	2 (9.5)
20+	1 (4.8)

### Technical aspects of telemedicine

Both patients and providers reported high degree of satisfaction with telemedicine overall (83.3%; 71.4%), convenience (94.4%; 85.7%), and having adequate time during the visit (94.4%; 76.2%), and the majority would recommend telemedicine to others (94.4%; 95.2%). Patients reported telemedicine saved them time (88.9%) and that all of their issues were addressed (83.3%; Table 3). Telemedicine platforms were integrated into electronic health system for 14 (66.7%) providers, and the majority never or almost never (13/21) had problems with logging on. Multiple platforms were used by nine providers.

**Table 3.** Technical Aspects of and Satisfaction with Telemedicine Visits

Variable	Patient (***n*** = 18), ***n*** (%)	Provider (***n*** = 21), ***n*** (%)	Both (***n*** = 39), ***n*** (%)
Overall satisfaction (experience)	15 (83.3)	^a^15 (71.4)	30 (76.9)
Convenience	17 (94.4)	^a^18 (85.7)	35 (89.7)
Saving time	16 (88.9)	^a^11 (52.3)	27 (69.2)
Issues addressed	15 (83.3)	^a^13 (61.9)	28 (71.8)
Adequate time	^a^17 (94.4)	^b^16 (76.2)	33 (84.6)
Access (can chose more than 1)			
Cellphone-audio only	2 (11.1)	4 (19.0)	6 (15.4)
Cellphone-audio and video	10 (55.6)	9 (42.9)	19 (48.7)
Computer/laptop	5 (27.8)	19 (90.5)	24 (61.5)
iPad/tablet	1 (5.6)	4 (19.0)	5 (12.8)
Audio clarity	17 (94.4)	^a^16 (76.2)	
Visual clarity	^a^16 (88.9)	^a^16 (76.2)	
Technology support availability	^b^13 (72.2)	^a^13 (61.9)	26 (66.7)
Would recommend	17 (94.4)	^a^20 (95.2)	37 (94.9)

^a^ One missing.

^b^ Two missing.

### Lack of in-person examination components

Lack of in-person examination components were of more concern to providers than patients, including height/weight measurement (*p* < 0.001), vitals (*p* < 0.001), and glycated hemoglobin (HbA1c; *p* < 0.001). Lack of physical examination was not significant (*p* = 0.063; Table 4).

**Table 4.** Concerns with Lack of In-Person Examination Components

	Patient mean	Provider mean	***p***
Height/weight	1.22	3.25	<0.001
Vitals	1.28	2.45	<0.001
Physical examination	1.72	2.30	0.063
A1c	1.72	3.50	<0.001

### Telemedicine endocrine care

Of the nine patients with CFRD, four were able to download their glucometer and three reported that it was easy to do so. When providers were asked to compare treating patients with CFRD to those with type 1 diabetes (T1D), there were no differences in availability of meter, continuous glucose monitor (CGM), or pump download, and no difference in concerns regarding the lack of data from these devices.

### Themes from qualitative questions

Both patients and providers were asked a series of open-ended questions regarding their experiences with telemedicine. Patient questions covered technological issues, what was easiest and added by telemedicine, what was hardest and missed by telemedicine, and what could have made the experience better. Overall, patients found telemedicine visits easy to attend with minimal technological problems. Many patients liked not having to travel to the appointment and the reduced risk of infection with telemedicine. Patients did miss having personal interactions and labs completed at the time of an in-office visit. Survey questions, themes, and quotations are listed in Table 5.

**Table 5.** Themes from Patient Qualitative Questions

Why did you not download your glucometer?	**No clear theme was identified among responses** “I did not have the equipment to do the download” “I provided 10 days of my glucose log by phone”
What was the hardest part of telemedicine?	**Overall, most patients did not have any problems. Those that did have difficulty mainly complained about technology problems.** “I'm not technically inclined, but nothing specifically was too hard” “Nothing was too difficult” “The voice connection wasn't good at first but it was fixed during the visit”
What was the easiest part of telemedicine?	**Not having to drive, avoiding an office visit due to COVID-19 concerns** “Not having to drive 2.5hrs to get to the appointment” “I didn't have to leave work and was able to complete the visit easily” “Not having to drive and worry about getting sick in the waiting room” “It was quick” “The software was easy to use”
What do you feel was missed by having a telemedicine visit?	**Not getting HbA1c or other labs completed, lack of personal interaction** “My hgbA1c was not checked” “The lack of examination” “Not getting blood work done”
What do you think was added by having a telemedicine visit?	**Being safe from COVID-19, no travel time** “I was able to communicate with the doctor without traveling” “Lack of exposure during COVID-19 pandemic” “How quickly the telemedicine visit went”
What would make your telemedicine visit better?	**Most patients did not have any input** “I felt it was the best it could be” “I don't think anything, it saved me time during the middle of a work day” “An easier way to schedule it, and better timing of the appointment”

Common themes of patient responses are in BOLD, with quotes from patient responses listed.

HbA1c, glycated hemoglobin.

Provider questions included what was easiest and added by telemedicine, what was hardest and missed by telemedicine, perceived differences in treating patients with T1D compared to those with CF by telemedicine, and what could make telemedicine better. Issues with technology/connection as well as missing vitals, labs, glucose data, and physical examination were common concerns. A decrease in the rate of appointment “no shows” and improved convenience for patients and their families with improved access to care were noted advantages. Survey questions, themes, and quotations are listed in Table 6.

**Table 6.** Themes from Provider Qualitative Questions

What is the hardest part of telemedicine visits?	**Technical/connection issues, not having all data information (labs, downloads)** “If there are technical difficulties, by the time they are resolved, the visit time is over. Patients are frustrated about the “check in process” since if we are running behind, they are just waiting with a blank screen.” “Not always having the necessary information at the time of the visit, poor connection/technical issues” “Technical issues from patient side. Not having all information: if patients do not know how to download their meters/pumps/sensors then it is an issue”
What is the easiest part of telemedicine visits?	**Decreased rates of no shows, convenience, convenience especially for those that are far away/rural area** “Scheduling! It's so easy for patients to be seen. I'm able to see patients now who haven't been seen in forever due to issues with scheduling and coming in to Boston. I'm also able to see patients I'm worried about more often because they don't have to take hours out their day to come in in-person.” “In many ways its more personal seeing kids in their homes. They show you their rooms, their pets, their artwork. It's great for the dietitian, she goes through their cupboards and fridge with them.”
What do you feel is missed by having a telemedicine visit?	**Lack of physical exam/face to face interaction, POCT A1c/labs, lack of care from educators/multidisciplinary care** “Lack of a physical exam, which particularly in pediatrics (ex. puberty exam), may affect management; lack of A1c and ability to get labs the day of the visit” “Human connection for those who are struggling, and lack of ability to review data on those who are not engaged with their care” “Foot exams. establishing rapport for new patient visits. helping patients with glucose data downloads”
What do you think is added by having a telemedicine visit?	**Convenience, better compliance with visits, convenience for patients that live far away, improved access to care** “Convenience, attendance at clinic appointments is improved, patients who provide glucose data feel they have received equivalent care with greater convenience” “It made care more accessible to patients especially those who live far and have transportation issues. We are now able to do a faster follow up for those with uncontrolled glucose control.” “Access to care. Lower infection risk.”
What are the differences in providing telemedicine care to patients with T1D compared to those with CF?	**Lack of lung exams/PFTs and weight for CF patients** “Weight and BMI tends to be important for CF patients. This is missing at Telemedicine visits.” “Not much! Many of my CF patients are very medically savvy and so telemedicine seems less overwhelming for them” “Not much different in terms of providing telemedicine care. but I find it more important to track weight for example in patients with CF. also due to COVID they are not always doing spirometry studies so this has been impacted as well.”
What would make telemedicine visits better?	**Having all data available at time of visit, better meters/pump/CGM downloads before visit, less technology/connection issues** “If patients had BP machines & scales at home and consistently uploaded CGM/pump/meters from home prior to the visit” “Support staff to assist with troubleshooting and downloading of information from devices, collected prior to the visit “ “Increased patient access to data/technology to complete the visits”

Common themes of provider responses are in BOLD, with quotes from provider responses.

CF, cystic fibrosis; CGM, continuous glucose monitor; PFTs, pulmonary function tests; POCT, point of care testing; T1D, type 1 diabetes.

## Discussion

The use of telemedicine has been studied in the care of patients with diabetes and CF; however, there is a lack of data regarding the care of patients with CFRD with telemedicine.^31^ To our knowledge, there are no studies assessing the satisfaction of CFRD and telemedicine from both the patient and provider perspectives. Our aim for this study was to gather data from both patients and providers regarding the endocrine care of patients with CF with telemedicine.

Overall, there was a high degree of both patient and provider satisfaction related to their telemedicine experience. Patients were more likely to report that telemedicine saved them time, which is not surprising as they did not have to travel to their appointment. When asked what the easiest part of telemedicine was and what could have been better, patients answered with the following:

“I didn't have to leave work and able to complete visit easily”

“Being able to do it via computer instead of driving to Syracuse”

There may have been extensive travel time for some of the patients who completed the survey. SUNY Upstate is one of 21 Cystic Fibrosis Care Centers in the state of New York according to the CF foundation website. The next closest centers to the west are in the cities of Rochester and Buffalo, which are about 90 miles and 150 miles away, respectively, from Syracuse. To the east, there is another center in Albany, which is about 150 miles away. This significant travel time saved does not apply to the providers since chart review, documentation, and many other tasks are not affected by telemedicine. However, this may be the reason for improved attendance rates as one provider stated the following:

“Convenience of not having the commute associated with the visit (families in particular) which, in my opinion, has resulted in better adherence/attendance”

The perceived improvement in “no show” rates was consistent with both patients and providers. Patients who may be used to travelling for hours to make appointments are probably more likely to complete a visit if it can be done from home. Patients can also avoid the added concern of exposing themselves to potential infections when coming into a physician office. As one patient stated the following about the easiest part of the telemedicine visit:

“Not having to drive and worry about getting sick in the waiting room”

Providers shared a similar sentiment regarding patient commutes and the rate of no shows when asked what the easiest part of the telemedicine visit was:

“Decreased no shows, convenient for patients who live far away”

An improvement in appointment visitation rates may be one of the most significant benefits to telemedicine. With the current shortage of endocrinologists, which is predicted to get worse in the coming years, utilizing the providers time more efficiently can benefit the entire health care ecosystem.^25^

One notable difference between patient and provider perspectives on telemedicine is with the lack of physical examination components. Providers were especially concerned about the lack of height and weight measurements along with the lack of HbA1c testing, as one provider mentioned the following:

“Weight and BMI tends to be important for CF patients. This is missing at Telemedicine visits.”

It was surprising that patients with CF rated the lack of physical examination components so low, as they are tied to outcomes with CF.^32^ One possible explanation for this difference in concern is that patients may have seen other providers for in-person visits. If patients had their physical examination components assessed at other times, they may not have been as concerned with the lack of physical examination components as it relates to their endocrine care. Patients with diabetes should have a fundoscopic eye examination and a comprehensive foot examination at least once yearly. They should also have their vitals measured at every appointment.^33^

Patients were not asked if they had physical examination components assessed as a part of their overall medical care. One solution to the issue of lack of physical examination may be to ensure that patients are seen at least once a year in person. Lack of laboratory testing was another area of concern when it came to telemedicine visits, as one provider stated the following about what was missed with telemedicine visits:

“Not always having a point of care A1c available for the clinic visit.”

Most patients used their phones for their visits, while most providers opted to use their computers/laptops. Although there was a high rate of satisfaction with the technical aspect of telemedicine, providers were more concerned about lack of technical support. Providers expressed frustration regarding missing data or not being able to connect to the patients in a timely manner. These frustrations are not surprising, as they can impact the effectiveness of care delivery as well as cause providers to fall behind on schedule, as one provider stated the following about the hardest part of telemedicine:

“Extra time needed to print and mail lab orders. Not having labs done before the visit or at the time of the appointment. Technical issues with patients.”

While telemedicine may have been new to many providers at the onset of the COVID-19 pandemic, almost all providers would recommend telemedicine use in the future. Over 60% of the provider respondents have been in practice for at least 5 years. This indicates a willingness to shift practice patterns to incorporate telemedicine in the future. There are several limitations to this study. The sample size of both patients and physicians was small. The patients surveyed were from only one endocrine practice and their experiences with telemedicine may not apply to the CF and CFRD population as a whole. In addition, only endocrinologists were surveyed for the provider viewpoint.

Most patients with CF are seen by an interdisciplinary team that includes other physician specialties, nurses, pharmacists, respiratory therapists, nutritionists, and social workers, as well as diabetes educators for patients with CFRD. More investigation is needed to understand the perspectives of not just physicians but also those of the entire medical team to better understand how it affects patient care delivery. Although telemedicine appears to have many advantages, it does not replace the traditional in-person office visit model. It cannot replace physical examinations and the convenience of obtaining laboratory work in the office. However, it allows for patients to get specialty care even from great distances.

## Conclusion

The COVID-19 pandemic caused a rapid transition to telemedicine. While the acuity of the pandemic has passed and more patients are now being seen for in-person office visits, some patients and providers are still using telemedicine. Both patients and providers have had the opportunity to experience both the conveniences and inconveniences that are associated with telehealth. To the authors' knowledge, this is the only study that has evaluated satisfaction with telemedicine related to endocrine care of patients with CF and CFRD. Further research is needed to explore management of CFRD by telemedicine and how telehealth will affect chronic care delivery in the post-pandemic landscape, as patients who require frequent visits for ongoing health issues may not want to give up the conveniences of telehealth.

## Authorship Contribution Statement

R.A. was responsible for survey administration, data collection, and writing. M.G. was responsible for statistical analysis, review, and editing. C.P.M. was responsible for statistical analysis. M.D. was responsible for conceptualization, writing, review, and editing.

## Abbreviations Used

CF: cystic fibrosis
CFRD: cystic fibrosis-related diabetes
HbA1c: glycated hemoglobin
REDCap: Research Electronic Data Capture
SD: standard deviation
T1D: type 1 diabetes
